# Nature-Based Therapeutic Intervention for Individuals with Post-Concussion Symptoms

**DOI:** 10.3390/bs14070594

**Published:** 2024-07-12

**Authors:** Sus Sola Corazon, Lisbeth Jul Olsen, Natasha Kæreby, Dorthe Varning Poulsen, Ulrik Sidenius, Stine Bekke-Hansen, Linda Marschner

**Affiliations:** 1 Department of Geosciences and Natural Resource Management, University of Copenhagen, 1958 Frederiksberg, Denmark; dvp@ign.ku.dk (D.V.P.); us@ign.ku.dk (U.S.); sbh@ign.ku.dk (S.B.-H.); 2 Municipality of Copenhagen, 2300 Copenhagen, Denmark; ti42@kk.dk; 3 Private Practitioner, 4800 Nykøbing Falster, Denmark; 4 Center for Rehabilitation of Brain Injury Denmark, 2300 Copenhagen, Denmark; linda.marschner@cfh.ku.dk

**Keywords:** nature-based health promotion, nature-based rehabilitation, nature-based therapy, health-promoting natural environments, nature activities, sensory stimulation, mild traumatic brain injury, mental fatigue

## Abstract

This study investigated the efficacy of a 10-session nature-based therapeutic intervention for people with post-concussion symptoms. The intervention involved physical and vestibular exercises, sensory training, relaxation, and psychoeducation, all of which were integrated with the natural environment in a forest therapy garden. This study was designed with a passive control period followed by the intervention (*n* = 30). The Mental Fatigue Scale (MFS) was the primary outcome measure. The secondary outcome measures were the Warwick–Edinburg Mental Wellbeing Scale and the short version of the Quality of Life after Brain Injury. A Likert scale was used to examine the mental strain of the sessions themselves. The MFS (primary outcome) exhibited a significant decrease with a medium-sized effect from before to after the intervention. The secondary outcomes exhibited significant increases from the beginning to the end of the intervention. All outcomes were sustained at follow-up ten weeks later. No significant difference was found from the control period. This study indicates that the described nature-based intervention is a feasible treatment for reducing prolonged post-concussion symptoms. However, it should be studied more in-depth to understand the impact of the natural environment and to validate the results on a larger representative population.

## 1. Introduction

Concussion, also known as mild traumatic brain injury, is a significant public health challenge affecting all population groups [1,2,3]. It has been found to have a once or more lifetime prevalence of 28.9% [2], although it is difficult to evaluate the exact extent, as many cases are assumed to go unreported [1].

Following a concussion, the majority recover within the first three months [4]. If the symptoms persist, which is estimated to be the case in 10–25% of reported concussions [5], it is diagnosed as post-concussion syndrome (PCS) according to the International Statistical Classification of Diseases and Related Health Problems (ICD-10) [6]. The diagnosis is based on the history of the head injury and reported symptoms. The prevailing cognitive and physical symptoms are mental fatigue, headaches, dizziness, problems with attention, concentration and memory, sensitivity to sensory stimuli, and sleep disturbances [7]. Emotional and behavioral changes are also common, such as irritability, emotional lability, sensitivity to stress, and, in more severe cases, anxiety and depression [8]. It can further lead to increased risk of social isolation and reduced quality of life [7,8]. On the socioeconomic level, PCS is related to prolonged sick leave, unemployment, and, in severe cases, early retirement [7,9,10]. Research has found the recovery prognosis to be best within the first year, but there is limited evidence for recovery hereafter [8,11].

### 1.1. Clinical Guidelines

There is no solid evidence base for the treatment of PCS, although research in the field is growing [12]. Danish clinical guidelines were published in 2021 and entailed the following seven recommendations for the non-pharmacological treatment of persistent concussion symptoms: early information, graded physical exercise, vestibular exercise, manual neck and back manipulation, psychotherapy, interdisciplinary treatment approaches, and oculomotor training [13]. The recommendations are based on a review and are in alignment with other international rehabilitation recommendations in the field [14]. The Danish review did not include present-moment awareness exercises, such as mindfulness-based stress reduction (MBSR), even though research has found some evidence in support of its effectiveness [15,16].

### 1.2. Enriched and Natural Environments

Enriched environments, defined as behavioral settings with good opportunities for physical and sensory stimulation, have been found to have potential in terms of rehabilitating traumatic brain injury [17,18,19]. Natural environments with a degree of biodiversity and wilderness can be seen as potentially enriched due to the diverse sensorimotor stimulation derived from seeing, hearing, touching, and smelling the living world and moving around on uneven surfaces. Therefore, the integration of the natural environment into the treatment of mild traumatic brain injury may have the potential to offer diverse opportunities for physical exercise and vestibular and sensory training, as recommended by the Danish treatment guidelines.

### 1.3. Attention Restoration

One well-established theoretical foundation for the mental health-promoting effects of natural environments is Attention Restoration Theory (ART), created by the psychologists Steven and Rachel Kaplan [20], which has found support in research [21]. According to ART, environments with diverse pleasant stimuli that provide a feeling of extent and of being away while being compatible with an individual’s needs can promote a state of soft fascination, which is also known as involuntary attention. In this effortless ‘drifting’ state, the mind can restore the time-limited directed attention resources. The potential of natural environments to promote mental restoration is very relevant in relation to post-concussion treatment, as rapid depletion of attention resources, overwhelming mental fatigue, and long recovery time are central symptoms [7,8]. However, it should be noted that not all natural environments promote restoration per se as they require certain qualities and characteristics to be perceived as such, while they must also be compatible with an individual’s needs and preferences [20,22].

### 1.4. Pilot Study

The potential of natural environments in restoring mental fatigue and providing sensorimotor stimulation led to the development of a pilot project to test an eight-session nature-based therapeutic intervention for individuals with post-concussion (*n* = 8). The results indicated a significant decrease in the participants’ post-concussion symptoms, and they reported experiencing nature-based exercises as meaningful and transferable to everyday life [23]. Additionally, they reported the natural environment as less mentally challenging than indoor rehabilitation settings, especially for social interaction. However, the small sample size and the lack of control conditions raise questions concerning the validity of the results. Therefore, a more extensive study was planned. 

### 1.5. Aim

The present study aimed to test the efficacy of a 10-session nature-based therapeutic intervention for post-concussion symptoms integrating physical and vestibular exercises, sensory training, relaxation, and psychoeducation with the natural environment. The effect of the intervention was measured in terms of change in post-concussion symptoms, well-being, and life quality.

## 2. Materials and Methods

### 2.1. Trial Design and Sample Size

In this study, the participants served as their own controls to examine whether recovery could occur spontaneously over time. Therefore, the participants were enrolled in a passive wait period equal in time to the following intervention.

A power analysis determined the minimum sample size of 26 persons (2-sample *t*-test: α = 0.05, 95% CI; expected s.d. = 3, β = 0.8; *p* < 0.05). It was decided to aim to recruit 40 participants, to account for dropouts. The size of the groups was determined to be a maximum of 10 participants due to experiences from a previous group intervention in nature [24].

### 2.2. Recruitment

The recruitment took place between the winter of 2022 and the summer of 2023. The opportunity to participate was advertised through several channels. The Danish Concussion Association and the Danish Center for Rehabilitation of Brain Injury, both partners in the project, included information about the intervention on their electronic news channels and in newsletters to members. Municipal brain injury coordinators in the Zealand region were also informed about the project and provided with recruitment material, which they distributed. Potential participants were encouraged to self-refer to the project by either email or phone to one of the responsible therapists conducting the intervention.

### 2.3. Inclusion

At the first contact with the therapists, potential participants were informed about the study, the content of the nature-based intervention, and their rights. If the person was still interested, the therapists conducted an initial screening based on the inclusion criteria. Afterward, a time for an interview and screening at the Danish Center for Rehabilitation of Brain Injury was agreed upon. A neuropsychologist at the Center held the meeting. This was also the first data collection point.

**Figure 1:**
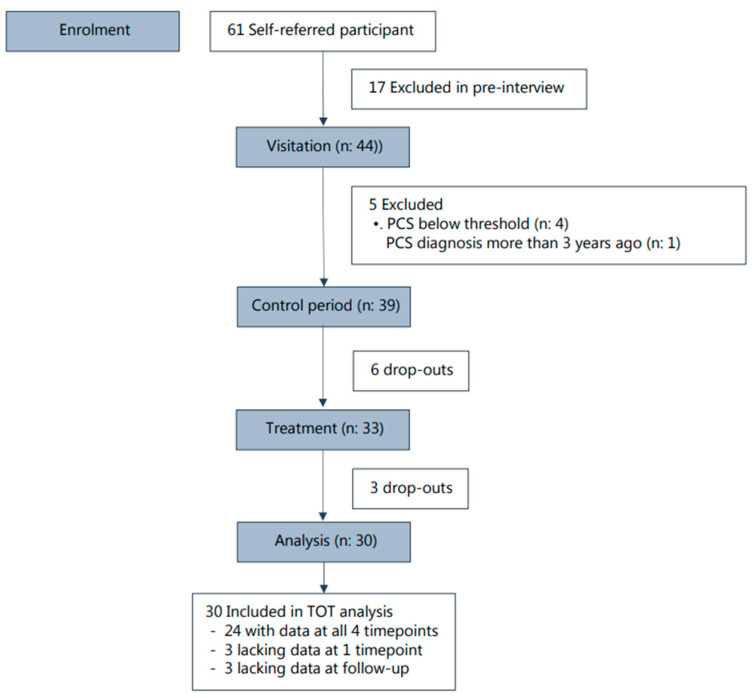
Participant flow diagram.

To be eligible, the participant had to have suffered the concussion within the previous three years and had to be experiencing post-concussion symptoms above the suggested cutoff score for mild to no symptoms measured by the Mental Fatigue Scale [25]. The participants could not receive any other treatments in the control or intervention period. They had to be above 18 years of age, be able to speak and understand Danish, and be able to transport themselves to the therapy garden for the sessions. Individuals with severe psychiatric morbidity, psychotic disorders, personality disorders, suicidal tendencies, and drug or alcohol problems were excluded, based on the Mini-International Neuropsychiatric Interview [26]. Of the 61 self-referred participants, 44 participants underwent the inclusion interview at the Center for Brain Injury, and subsequently, 39 participants were found eligible and enrolled in the study. Thirty participants completed the intervention (see participant flow diagram, Figure 1).

### 2.4. Nature-Based Intervention

The participants were divided into four treatment groups with two groups having weekly sessions in the spring of 2023 and two groups having weekly sessions in the autumn of 2023. The program consisted of ten 3 h sessions all of which took place at the University of Copenhagen’s therapy garden, Nacadia^®^.

#### 2.4.1. The Therapy Garden Nacadia

The garden consists of a 1.4-hectare semi-wild forest garden located within an arboretum containing the largest collection of different trees and shrubs in Denmark. The design of the garden is based on the evidence-based health design model within landscape architecture (EBHD) [27]. The garden includes areas with different characteristics and opportunities for activities. The sessions usually begin and end at an outdoor fireplace, which is surrounded by benches and shielded at the back by permanent stacks of firewood. From the fireplace, there is a view of a wild meadow, which has a large circle cut into the tall grass. The circle is used for physical activities such as stretching and yoga-inspired movements. A stream runs through the meadow and ends in a large pond. From the pond, there is a view of a lake, which is located just outside the therapy garden. The meadow is surrounded by a little forest, which enhances the feeling of privacy from the public arboretum. The forest is crossed by small paths and little secluded areas with benches. The lower end of the garden contains a wooden building for gardeners with a large raised wood veranda and a 100 m^2^ renovated greenhouse. The greenhouse is a mini version of the garden with a social meeting place, a wooden platform for physical exercise, and little spaces for privacy surrounded by climbing plants and bushes. The gardenerbuilding includes toilets and items to keep the participants warm and dry such as insulated overalls, boots, raincoats, sleeping bags, mats, blankets, and lambskins, which can be used in the garden. The items make it possible for the participants to be outside in the garden all year round.

#### 2.4.2. The Therapeutic Concept

The overarching therapeutic concept for the Nacadia therapy garden is built upon elements from cognitive behavioral therapy and present moment awareness approaches integrated with environmental psychology [28,29], especially attention restoration theory, which emphasizes soft sensory stimulation as a means of restoring fatigued cognitive resources [20].

An interdisciplinary team within the Nature, Health & Design research group at the University of Copenhagen developed the 10-session program. The team also included the therapists, a physiotherapist, and a nurse with a master’s in nature-based rehabilitation. The program integrated the national clinical recommendations and entailed graded physical and vestibular exercise, sensory-motor training, psychoeducation on symptom management, and relaxation. Relaxation was included to avoid depleting the participants’ resources during the sessions and to introduce them to tools to alleviate mental fatigue. The transferability of the different exercises to everyday life was emphasized by having homework between sessions.

#### 2.4.3. Structure and Content of the 10 Sessions

All sessions took place in the therapy garden and surrounding arboretum, integrating the activities and experiences found in the natural environment.

The sessions had the same overall structure. They started at the fireplace in the garden, where the therapist guided a seated present moment awareness exercise with a focus on sensing the body, the surroundings, and finally the group. This was followed by a discussion about experiences with the previous week’s home exercise. The aim of this gathering was to both enhance the feeling of community and reduce potential strain.

Afterward, the group went to the open circle in the meadow, where the therapist guided stretching exercises, which included vestibular training such as bending towards the ground and turning the body. The exercises included a focus on breathing with the movements to enhance bodily awareness. The participants were encouraged to monitor whether a movement became too challenging and alternative exercises were given throughout the exercises. The stretching program was expanded and slightly changed with new exercises added throughout the 10 sessions, as the participants became more familiar with them and were comfortable with challenging their individual threshold.

The stretching and vestibular training was followed by a break during which the participants could enjoy a moment’s rest alone in the garden, have a cup of tea, or be social.

The session continued with a more physically active exercise, which often included a walk around the arboretum with aerobic or ocular tasks. The group worked together, but individuals were given the opportunity to do more and less challenging options, for example, how quickly they could run from tree to tree, or how long they could engage in, for example, an ocular training session.

The exercises often led to some strain on the participants’ mental, physical, and social resources and, therefore, they were followed by some restorative alone time in the garden. Little tasks were given to promote the restoration such as finding a tree that seemed appealing and making themselves comfortable there. Blankets, mats, and sleeping bags were at the participants’ disposal. In some of the sessions, the alone time entailed a reflective exercise with questions the participants could answer in their journals such as ‘what gives you energy in your everyday life and what drains you?’.

The participants met again at the fireplace or at the circle in the meadow. A psycho-educative theme was presented by the therapists, often illustrated with a nature metaphor or mirroring in the natural environment. The theme was thereby given a physical dimension, which is thought to support understanding and learning [30,31]. For example, when talking about energy management or coping with everyday challenges related to post-concussion symptoms, the natural element fire could be included to illustrate ways of approaching difficult situations. Another example is likening the participants’ loss of abilities to a tree in a storm, which has to let go of branches to stay rooted.

The psycho-educative session was followed by a guided body scan and relaxation, which sometimes included the use of hot stones or eyebags, laying on mats on the ground in the garden and clothed according to the weather, or on the wooden platform in the greenhouse in inclement weather.

The session was concluded around the fireplace, where each participant was invited to share a few words on what had resonated with them and had been useful in the day’s session to strengthen their understanding of what worked for them. The next week’s home exercise, which drew on the content of the session, was presented.

A detailed overview of each session in the program is published online (in Danish) [32].

### 2.5. Data Collection

The participants completed the same self-rated questionnaire at four different points in time: at the inclusion interview (baseline), at the end of the control period (I start), at the end of the intervention (I end), and ten weeks later (follow-up).

The questionnaire entailed three validated scales: The Mental Fatigue Scale, the Warwick–Edinburgh Wellbeing Scale, and the Quality of Life after Brain Injury (QOLIBRI). The neuropsychologist helped the participants fill out the questionnaire at the inclusion interview, which also entailed background questions related to age, gender, socioeconomic and employment status, and use and preference of nature. The questionnaire was sent via email as an online link at the following three data points.

### 2.6. Primary Outcome Measure

The Mental Fatigue Scale (MFS) comprises 15 questions concerning affective, cognitive, and sensory symptoms and sleep [33]. It is scored on a seven-point scale from 0 to 3 with ½ intervals. Each whole number (0,1, 2, 3) has a verbal example attached. A rating of 0 corresponds to no symptoms and 3 to the maximum experienced symptoms. The scale has a guiding threshold score of 10.5 points, which can be interpreted as having none or mild symptoms. The MFS was chosen as the primary measure instead of the more widely used Rivermead Scale due to its depth in symptom description related to everyday functioning and focus on various aspects of mental fatigue, which is a prevailing long-term symptom [25]. On the other hand, the Rivermead Scale is more straightforward to fill out and includes more physical symptoms closely related to the immediate head trauma [34].

### 2.7. Secondary Outcome Measures

The Warwick–Edinburgh Wellbeing Scale (WEMWBS) measures overall mental well-being. The scale consists of 14 statements about positive mental well-being and psychological functioning [35]. The level of agreement with each statement is rated on a scale from 1 (never) to 5 (all of the time), which is summed to a total score between 14 and 70. The WEMWBS is widely used in health research [36].

As the name indicates, the Quality of Life after Brain Injury (QOLIBRI) was specifically developed to measure an individual’s quality of life following a brain injury [37]. The short version of the QOLIBRI Overall Scale consists of six statements concerning quality of life across four domains: physical, cognitive, emotional, and social relationships [38]. The level of agreement with each statement is rated on a scale from 1 (not at all) to 5 (very much), which is summed and converted to a score between 0 and 100.

### 2.8. Likert Scales on Session Level

A Likert scale was used to investigate whether participating in the single sessions increased mental fatigue. The scale was filled out at the beginning and at the end of each session. The score ranged from 0 (total mental drain) to 10 (total mental energy). Likewise, possible changes in mood from the beginning to the end of each session were examined by use of a Likert scale, where 0 signified the lowest level, and 10 was the highest positive mood.

### 2.9. Statistical Analysis

The data were analyzed per the protocol and only included the individuals who had completed the intervention to determine the effect of the treatment only on the treated (TOT). This approach excluded the dropouts who underwent the control period but not the intervention. To determine whether the control period would show any significant difference if all enrolled participants were included, an intention-to-treat analysis (ITT) was performed for the control period, which showed no significantly different results from the TOT approach.

Due to the format of the online questionnaire, all questions had to be answered before it could be submitted. Therefore, no single responses were missing in the outcome measures or the subsequent data analysis.

The Shapiro–Wilk test showed no significant deviations from the normal distribution for the primary and secondary outcomes. Therefore, the following statistical analysis was based on a normal distribution sample. A paired sample *t*-test was applied between two subsequent data points. Effect sizes were calculated using Cohen’s d for dependent samples. Pearson’s r-correlation test was used to determine whether there was any correlation between changes in primary and secondary outcomes.

The data from the two Likert scales both showed significant differences from the normal distribution on the Shapiro–Wilks test. Therefore, a Wilcoxon signed rank test was applied to these outcomes to test for significant differences in pre–post session outcomes.

## 3. Results

### 3.1. Baseline Characteristics

The baseline characteristics of the participants (*n* = 30) are presented in Table 1. Most participants were women (*n* = 23), held a university degree, and were middle-aged. The employment status revealed that there was a preponderance of participants on part/full-time sick leave or participating in municipal subsidized work trials to which people are usually referred after long-term sick leave and unemployment. When they started the project, most of the participants had been suffering from post-concussion symptoms for one year or longer. Only two participants reported that the injury had occurred within the same year. The participants had all previously engaged in a range of rehabilitation initiatives including physiotherapy, massage, oculomotor training, medication, and sessions with neuropsychologists and psychotherapists.

The participants were asked to rate their enjoyment of nature, use of natural environments, and perceived benefits (Table 2). The results clearly show a strong positive inclination towards nature and a high degree of self-perceived positive benefits derived from frequent use.

### 3.2. Primary Outcome: The Mental Fatigue Scale

The results on the Mental Fatigue Scale (MFS) showed no significant difference from the control period (*p* > 0.01), indicating that the symptoms did not spontaneously change (Table 3). This supports previous research on limited spontaneous recovery from PCS after the initial period [8,11]. The intervention period showed a significant decrease in the MFS score (*p* < 0.01) with a moderate effect size (r = 0.55) [39]. The participants’ mean score was still above the suggested 10.5 point threshold indicating no to mild symptoms [25].

**Figure 2:**
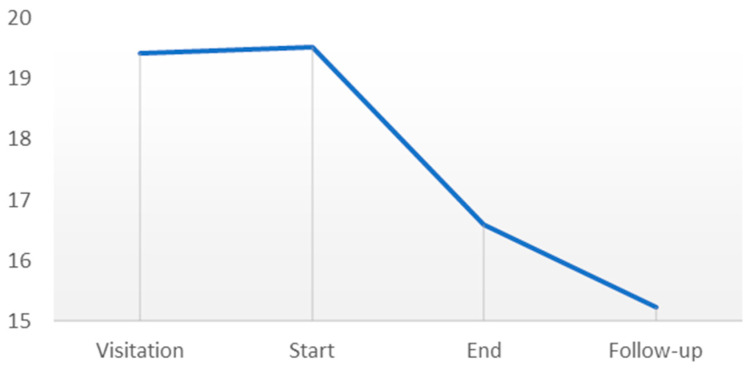
Visual representation of the results from The Mental Fatigue Scale.

No significant difference was found in the follow-up period, indicating that the decline in symptoms during the intervention remained persistent ten weeks after its conclusion. A continuous small decline is seen, although it is not significant (Figure 2).

### 3.3. Secondary Outcomes

A significant difference was found on the Warwick–Edinburgh Mental Wellbeing Scale between the start and the end of the intervention, approaching a medium-sized effect (*p* < 0.01, d = 0.45). No significant effect was found for either the control (*p* > 0.01) or follow-up period (*p* > 0.01) (Table 4). The change in the mean of 3.48 points from the start to the end of the intervention is above the minimal clinically important level of change (>3) [35]. The mean of 47.0 is still below that of the general population (51.0) based on a representative sample from the United Kingdom [36].

The results for the Quality of Life after Brain Injury Scale (QOLIBRI_OS) are weaker than the two previous outcomes (Table 5). It shows a small significant difference from the intervention period (*p* < 0.05, d = 0.34) and no significant difference from the control (*p* > 0.05) or follow-up period (*p* > 0.05). The QOLIBRI guidelines do not entail a threshold score for significant change.

### 3.4. Correlation between Primary and Secondary Outcomes

The Pearson’s r correlation test revealed a significant moderate negative correlation (*p* < 0.05, r = −0.559) between the decline in the participants’ scores in the MFS and the increase in their scores in WEMWES from the beginning to the end of the intervention, and likewise a significant but weak negative correlation (*p* < 0.05, r = −0.378) between the MFS and QOLIBRI-OS scores. Overall, this indicates a tendency towards a decline in post-concussion symptoms to correlate with an improvement in mental well-being and health-related quality of life. However, no causal relationship can be determined.

### 3.5. Likert Scale Outcomes from Single Sessions

Attendance at the single sessions was high, with 80% (*n* = 24) of participants attending between 8 and 10 sessions. The minimum required attendance was 50%.

The results of the Wilcoxon Signed-Rank test revealed a significant difference with a medium-sized effect in terms of self-perceived mental fatigue (Z = 4, r = 0.351, *p* < 0.0001) with a mean increase of 0.52 points in mental energy (Table 6) from before to after a single session. A significant difference with a large-sized effect in terms of self-assessed positive mood (Z = 6,5, *p* < 0.001) with a mean increase of 0.92 points (Table 6) was also found from before to after single sessions.

## 4. Discussion

The participants in the study had been suffering from persistent concussion symptoms for three months to three years, with the majority suffering for more than one year, which, according to research, diminishes the prospect of spontaneous recovery [8,11]. As there is little evidence-based consensus regarding the appropriate treatment of post-concussion syndrome [13], the participants had tried a range of public and private treatments previously. At the beginning of the intervention, their mean self-reported quality of life was markedly below the value of 52, which is recognized as indicating low or impaired quality of life after brain injury [40]. However, after the intervention, the participants’ mean score for life quality was approaching 50. Additionally, the participants’ self-assessed mental well-being was increased significantly by the intervention. Even though most participants had been suffering from concussion symptoms for more than a year, the results in terms of the Mental Fatigue Scale indicate that participation in the intervention reduced the symptoms. However, it should be noted that even though the symptoms decreased significantly, the mean value at the end of the intervention was still at the low end of the range of what is considered to indicate moderate symptoms [25]. Still, the positive results highlight that change is possible, and they can be seen as an argument for providing rehabilitation initiatives even years after the occurrence of the concussion incident itself, as persistent concussion symptoms are known to increase the risk of mental co-morbidities besides lowering life quality [7,8,9,10].

The therapy garden and surrounding arboretum added value to the sessions in several ways besides potentially promoting mental restoration, as proposed in the attention restoration theory [20]. The diversity of vegetation, different sloping terrains, and characteristics from dense forests to meadows and streams provided the therapists with a vast range of physical and sensory exercises. The length and duration of the walks on flat or sloping terrain could be varied, the ocular exercises could be focused on a small movement like in the stream or larger movements and longer distances at the lake, and the restful moments could take place in the same environment by simply changing the instructions. According to the results on the Likert scale, the balance between exercise and rest was successful, thereby avoiding mental drain, which may otherwise be a hindrance in itself to participating in rehabilitation initiatives.

### Limitations

The validity of the results is weakened by not having an active control group, for example, an indoor intervention. The results could have been further strengthened by the inclusion of neuropsychological tests in the effect measures.

With the current research design, it is also not possible to determine the impact of the natural environment on the intervention. This is a general limitation of complex health interventions to evaluate the significance of single factors in the integrated whole.

Gender, age, and the preference for spending time in nature can be seen as limitations in generalizing the results, with an overrepresentation of women, participants above 40 years, and a strong preference for spending time in nature.

## 5. Conclusions

This study indicates that the described nature-based intervention is a feasible treatment for reducing symptoms and improving mental well-being and potentially also life quality for people with long-term post-concussion symptoms. The role of the natural environment in the intervention needs to be studied in more detail, preferably with an active control group receiving a comparable indoor rehabilitation to better understand its significance.

The results suggest that rehabilitation may facilitate a reduction in post-concussion symptoms even years after the injury has occurred, and, further, that it is possible to modulate the rehabilitation sessions alternating between activity and rest to avoid causing unnecessary mental fatigue.

## Figures and Tables

**Table 1:** Participants’ baseline characteristics (*n* = 30 *).

Gender: Female/Male, *n*	23/7
Age, *n*	
20–29	2
30–39	4
40–49	7
50–59	10
60–69	3
70–79	3
Education, *n*	
Primary/secondary school	2
Vocational training	3
University bachelor’s degree	12
University master’s degree	11
Employment status *n*	
Full-time employed	2
Reduced time/Part-time sick leave	5
Full-time sick leave	7
Unemployed	1
Student	2
Retired	4
Municipal subsidized employment	7
(job trial, internship, flex job)	
Concussion incident	
2019	3
2020	10
2021	10
2022	5
2023	2

* The data from the background questionnaires included missing responses to single questions. Therefore, the sum of responses for the variables does not match the total number of participants.

**Table 2:** Participants’ use and perceived benefits of nature (*n* = 30 *).

Do you enjoy being in nature?	
Very much	29
To some extent	0
Somewhat	0
To a low extent	0
Not really	0
How often do you spend time in nature?	
Every day	19
A few times a week	8
Once a week	2
Once a month or more	0
Rarely	0
To what degree do you consider the time spent in nature to benefit…	
Your physical health?	
Very much	
To some extent	19
Somewhat	10
To a low extent	0
Not really	0
Your mental health	
Very much	25
To some extent	4
Somewhat	0
To a low extent	0
Not really	0
	0

* The data from the nature use questionnaire entailed missing responses from one participant.

**Table 3:** Results on the Mental Fatigue Scale.

Data Points	Mean	SD	*p* Value *	Cohen’s d
Visitation	19.42	3.21		
Control period				
Start	19.52	4.41	0.525	NA
Intervention period				
End	16.59	6.06	0.005	0.553
Follow-up	15.83	6.71	0.544	NA

* Between two consecutive data points. NA: Not applicable.

**Table 4:** Warwick–Edinburgh Mental Wellbeing Scale.

Data Points	Mean	SD	*p* Value *	Cohen’s d
Visitation	45.00	6.55		
Control period				
Start	43.62	7.54	0.094	NA
Intervention period				
End	47.00	7.43	0.006	0.452
Follow up	47.84	8.98	0.676	NA

* Between two consecutive data points. NA: Not applicable.

**Table 5:** Quality of life after brain injury (QOLIBRI-OS).

Data Points	Value%	SD	*p* Value *	Cohen’s d
visitation	39.74	18.68		
Control period				
Start	41.24	20.42	0.958	NA
Intervention period				
End	48.56	22.67	0.012	0.339
Follow up	49.36	26.21	0.879	NA

* Between two consecutive data points. NA: Not applicable.

**Table 6:** Pre- and post-single-session scores.

Data Points	Mean	SD	*p* Value	Pearsons r
Level of mental fatigue				
Pre-session	4.60	1.84		
Post-session	5.12	2.02	0.000	0.351
Level of well-being				
Pre-session	5.08	1.81		
Post-session	6.01	1.95	9.029 × 10^−11^	0.564

## Data Availability

Data are unavailable due to the General Data Protection Regulation restrictions on sharing. Permission to use the research data was solely granted to the named researchers involved in this study at the Faculty of Science, University of Copenhagen, Denmark. The data collection was approved by the Faculty board of SCIENCE, UC, Denmark (Protocol no.2 901532/4242).
